# Hypoxia-Induced Insulin Resistance Mediates the Elevated Cardiovascular Risk in Patients with Obstructive Sleep Apnea: A Comprehensive Review

**DOI:** 10.31083/j.rcm2506231

**Published:** 2024-06-25

**Authors:** María M. Adeva-Andany, Alberto Domínguez-Montero, Elvira Castro-Quintela, Raquel Funcasta-Calderón, Carlos Fernández-Fernández

**Affiliations:** ^1^Internal Medicine Department, Hospital General Juan Cardona, 15406 Ferrol, Spain

**Keywords:** metabolic syndrome, hypertriglyceridemia, visceral obesity, non-alcoholic fatty liver disease, essential hypertension, diabetes, chronic kidney disease, cardiovascular risk, tissue hypoxia

## Abstract

Patients with obstructive sleep apnea (OSA) experience insulin resistance and its clinical consequences, including hypertriglyceridemia, reduced high density lipoprotein-associated cholesterol (HDL-c), visceral adiposity, hepatic steatosis, increased epicardial fat thickness, essential hypertension, glucose intolerance, increased risk for type 2 diabetes, chronic kidney disease, subclinical vascular damage, and increased risk for cardiovascular events. Obesity is a major contributor to OSA. The prevalence of OSA is almost universal among patients with severe obesity undergoing bariatric surgery. However, insulin resistance and its clinical complications occur in OSA patients irrespective of general obesity (body mass index). In OSA patients, apnea episodes during sleep induce oxyhemoglobin desaturation and tissue hypoxia. Insulin resistance is an adaptive response to tissue hypoxia and develops in conditions with limited tissue oxygen supply, including healthy subjects exposed to hypobaric hypoxia (high altitude) and OSA patients. Indicators of oxyhemoglobin desaturation have been robustly and independently linked to insulin resistance and its clinical manifestations in patients with OSA. Insulin resistance mediates the elevated rate of type 2 diabetes, chronic kidney disease, and cardiovascular disease unexplained with traditional cardiovascular risk factors present in OSA patients. Pathophysiological processes underlying hypoxia-induced insulin resistance involve hypoxia inducible factor-1 upregulation and peroxisome proliferator-activated receptor-gamma (PPAR-γ) downregulation. In human adipose tissue, PPAR-γ activity promotes glucose transport into adipocytes, lipid droplet biogenesis, and whole-body insulin sensitivity. Silencing of PPAR-γ in the adipose tissue reduces glucose uptake and fat accumulation into adipocytes and promotes insulin resistance. In conclusion, tissue hypoxia drives insulin resistance and its clinical consequences in patients with OSA, regardless of body mass index.

## 1. Introduction 

**Fig. 1.**
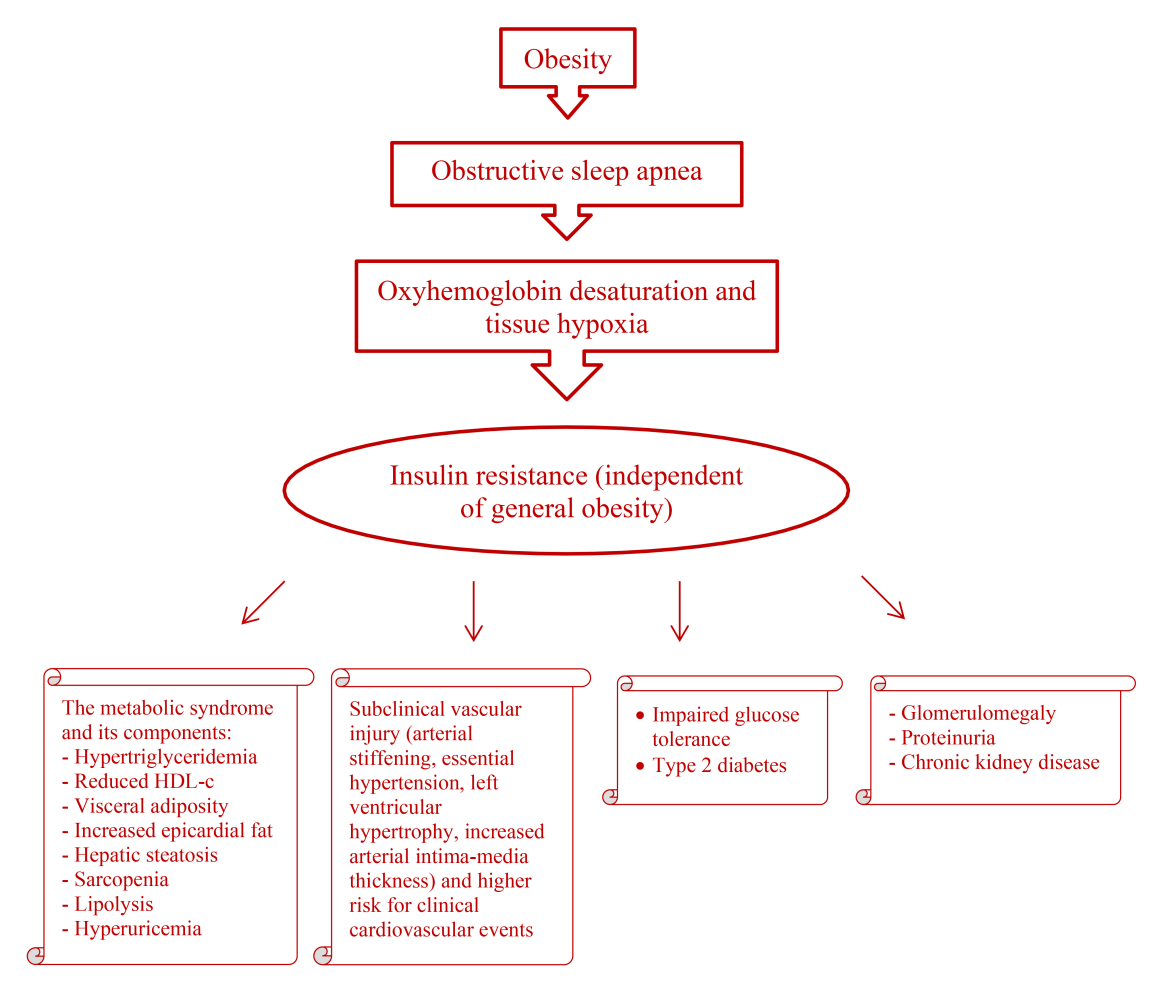
** Obstructive sleep apnea induces oxyhemoglobin desaturation and tissue hypoxia.** Insulin resistance develops as an adaptive process due to tissue hypoxia. Obesity contributes to obstructive sleep apnea, but insulin resistance and its complications (the metabolic syndrome and its components, type 2 diabetes, cardiovascular disease, and kidney disease) occur in patients with obstructive sleep apnea irrespective of general obesity. HDL-c, high density lipoprotein-associated cholesterol.

Patients with obstructive sleep apnea (OSA) typically suffer from insulin resistance and its clinical manifestations, such as metabolic syndrome, impaired glucose tolerance, reduced cholesterol associated with high density lipoprotein (HDL-c), hypertriglyceridemia, visceral adiposity, non-alcoholic fatty liver disease (NAFLD), increased epicardial fat thickness, subclinical vascular damage including arterial stiffening and increased arterial intima-media thickness (IMT), essential hypertension, glomerulomegaly, and albuminuria. Eventually, clinical complications of insulin resistance may develop, such as type 2 diabetes (T2D), cardiovascular disease (CVD), and chronic kidney disease (CKD) [1, 2, 3, 4, 5]. Obesity is a major determinant of OSA occurrence. OSA prevalence in obese patients undergoing bariatric surgery is strikingly high, having been reported to be 81.1% [6] and 90.07% [7]. However, insulin resistance and its clinical consequences develop in OSA patients irrespective of general obesity. Insulin resistance in OSA patients is an adaptive response to oxyhemoglobin desaturation and tissue hypoxia due to nocturnal apnea episodes. Oxyhemoglobin desaturation has been strongly and independently associated with insulin resistance and its complications in patients with OSA. Both obese and non-obese OSA patients develop this metabolic adaptation (Fig. 1). Molecular mechanisms underlying hypoxia-induced insulin resistance in OSA patients involve hypoxia inducible factor (HIF) upregulation and peroxisome proliferator-activated receptor-gamma (PPAR-γ) downregulation, which reduces glucose uptake into adipocytes, suppresses adipogenesis, and causes whole-body insulin resistance [1, 2, 8, 9, 10, 11].

Information on the connection between hypoxia-induced insulin resistance and its clinical consequences (including CVD, CKD, and T2D) in patients with OSA may help improve the prevention and clinical management of these common conditions in clinical practice. A recent systematic review and meta-analysis that included 9 randomized studies reveals that nocturnal oxygen therapy reduces OSA severity compared to sham, suggesting that this therapy may facilitate the prevention of OSA complications [12].

## 2. Objectives

Short term exposure to hypobaric hypoxia (high altitude) induces adaptive insulin resistance and a metabolic adjustment that matches reduced oxygen delivery to mitochondrial oxygen consumption. As patients with OSA experience chronic nocturnal oxyhemoglobin desaturation and tissue hypoxia, we investigated the metabolic adaptation that takes place among these patients and its clinical consequences.

## 3. Methods

This is a comprehensive narrative review pertaining to insulin resistance in patients with OSA. A thorough and exhaustive search of published literature on this topic was performed by using the PubMed database from its inception to February 2024. Only articles written in English and concerning human beings were included. Authors MAA, ADM, ECQ, and RFC contributed to the literature search. MAA approved the final list of included studies.

## 4. Obstructive Sleep Apnea is Associated with Insulin Resistance, its Clinical Manifestations, and its Clinical Complications

### 4.1 Obstructive Sleep Apnea is Associated with Insulin Resistance

Compared to control subjects, both children and adults with OSA experience insulin resistance, assessed by a variety of procedures including hyperinsulinemic euglycemic clamps. The prevalence of insulin resistance among OSA patients has been reported to be 64.3% [13]. Cross-sectional studies have observed an independent association between OSA and elevated homeostasis model assessment of insulin resistance (HOMA-IR) values [2, 3, 4, 5, 7, 13, 14, 15, 16, 17, 18, 19, 20, 21, 22, 23, 24, 25], fasting hyperinsulinemia [2, 3, 4, 14, 19, 21, 26, 27, 28], the triglyceride-glucose index, calculated as: In [fasting triglycerides (mg/dL) × fasting glucose (mg/dL)/2] [10, 11], and insulin resistance determined by intravenous glucose tolerance tests [29] or steady-state plasma glucose and insulin levels [27, 30]. The cross-sectional association between OSA and insulin resistance is independent of body mass index (BMI) and other confounding factors. OSA patients endure more severe insulin resistance compared to control individuals with comparable BMI. In addition, there is a positive correlation between OSA severity and intensity of insulin resistance (HOMA-IR index). Likewise, the prevalence of insulin resistance (evaluated by the HOMA-IR index and fasting insulin level) is higher among women with polycystic ovary syndrome and OSA compared to women with polycystic ovary syndrome without OSA, after controlling for BMI and other factors. OSA severity is highly correlated with the degree of insulin resistance among these patients [31]. Longitudinal trials establish that OSA precedes the development of insulin resistance. In a prospective community-based study that followed 141 non-diabetic patients with OSA for a mean period of 11 years, a diagnosis of OSA at baseline was independently related to the development of new-onset insulin resistance (evaluated by the HOMA-IR index and oral glucose tolerance tests) at follow-up, after controlling for BMI and other confounders. OSA independently increases the risk of insulin resistance [32]. Systematic reviews and meta-analyses confirm an association between OSA and insulin resistance determined via an elevated HOMA-IR [33] or the atherogenic index of plasma, calculated according to the formula: log (serum triglyceride level/serum HDL-c level) [34]. Compared to usual care, OSA therapy with continuous positive airway pressure (CPAP) [35, 36, 37, 38, 39, 40] or implantation of a mandibular advancement device [41], improves insulin resistance without changing BMI, supporting a role for tissue hypoxia in the pathogenesis of insulin resistance.

### 4.2 Obstructive Sleep Apnea is Associated with the Metabolic Syndrome and its Components

Correspondingly with the greater degree of insulin resistance, OSA is independently associated with clinical manifestations of this metabolic adaptation to hypoxia, such as metabolic syndrome and its components.

#### 4.2.1 Obstructive Sleep Apnea is Associated with the Metabolic Syndrome

Observational studies [4, 14, 16, 17, 18, 22, 42, 43, 44, 45, 46, 47, 48, 49, 50, 51, 52, 53, 54, 55, 56, 57] and a systematic review and meta-analysis [58] Consistently reveal that the prevalence of metabolic syndrome is increased in OSA patients, compared to control subjects, independently of BMI and other covariates. Metabolic syndrome is approximately 9 times more likely to occur in patients with OSA compared to control subjects. The number of features of metabolic syndrome increases with an increase in OSA severity, regardless of BMI.

#### 4.2.2 Obstructive Sleep Apnea is Associated with Increased Visceral Fat 

OSA patients (children and adults) suffer from marked visceral adiposity compared to control subjects, regardless of general obesity (BMI). The excess of visceral fat in OSA patients is determined by ultrasonography, magnetic resonance imaging, bioimpedance analysis, increased waist/hip ratio, widened waist circumference, or the visceral adiposity index (calculated with a formula that includes waist circumference, BMI, triglycerides, and HDL-c). Irrespective of body weight or BMI, OSA patients exhibit increased visceral adipose tissue, reflecting insulin resistance. In addition, OSA severity is strongly predictive of the visceral fat depot, unlike BMI, total fat, or subcutaneous fat [20, 22, 28, 43, 45, 50, 51, 52, 55, 56, 57, 59, 60, 61, 62, 63, 64, 65, 66, 67].

#### 4.2.3 Obstructive Sleep Apnea is Associated with Non-Alcoholic Fatty Liver Disease 

NAFLD is a clinical expression of insulin resistance. Therefore, the prevalence of NAFLD is higher in OSA patients compared to control subjects, independent of BMI and other confounding variables. Even in the absence of obesity, the prevalence of NAFLD is increased in OSA patients [7, 15, 68, 69, 70]. NAFLD is present in 81.8% of unselected OSA patients and 96.0% of patients with severe OSA undergoing bariatric surgery [69]. Patients with severe OSA are approximately 53 times more likely to have NAFLD compared to control subjects. There is a positive correlation between the severity of OSA and the degree of hepatic steatosis [70]. Systematic reviews and meta-analyses reveal that OSA is independently related to the development and progression of NAFLD determined by elevated liver enzymes and histological alterations including hepatic fibrosis, both in children and adults [71, 72, 73, 74].

#### 4.2.4 Obstructive Sleep Apnea is Associated with Increased Epicardial Fat Thickness

Increased epicardial fat thickness is an indicator of excessive visceral fat and consequently reflects the presence of insulin resistance. OSA patients show broader epicardial fat thickness compared to control subjects, independent of general obesity (BMI). Both non-obese and obese OSA patients have thicker epicardial fat compared to control individuals. In addition, OSA severity correlates with the magnitude of epicardial fat expansion [50, 54, 75, 76, 77]. These findings are confirmed in a systematic review and meta-analysis that included 9 studies and 1178 subjects [78].

#### 4.2.5 Obstructive Sleep Apnea is Associated with Insulin Resistance-Associated Dyslipidemia

Cross-sectional investigations show an association between OSA and insulin resistance-related dyslipidemia (hypertriglyceridemia and reduced HDL-c). Both adults and children with OSA endure a greater prevalence of hypertriglyceridemia and decreased HDL-c compared to control subjects, after controlling for covariates. Consequently, the triglyceride/HDL-c ratio and its logarithmic transformation (the atherogenic index of plasma) are higher in OSA patients, compared to control subjects. The association of OSA and insulin resistance-mediated dyslipidemia is independent of BMI and therefore occurs in non-obese as well as obese OSA patients. OSA severity is associated with worse lipid abnormalities (decreased HDL-c and increased triglyceride levels) [4, 5, 14, 20, 21, 25, 46, 54, 79, 80, 81, 82, 83]. In contrast, no independent association between OSA and serum levels of total cholesterol or low densitiy lipoprotein-associated choleserol (LDL-c) has been observed [79]. These findings are confirmed in a systematic review that pooled 25 studies for meta-analysis [84].

### 4.3 Obstructive Sleep Apnea is Associated with Subclinical Vascular Injury and Clinical Cardiovascular Events

It has long been known that patients with OSA endure a high cardiovascular risk that cannot be justified by conventional cardiovascular risk factors. OSA is independently associated with subclinical vascular injury (increased arterial stiffness, left ventricular hypertrophy, increased intima-media thickness, and reduced flow-mediated vasodilation), essential hypertension, and clinical cardiovascular events (coronary artery disease, congestive heart failure, peripheral artery disease, and stroke). Subclinical vascular injury and elevated cardiovascular risk in OSA patients have been regularly associated with insulin resistance, similar to subjects from the general population and other population groups, such as patients with diabetes and patients with CKD.

#### 4.3.1 Obstructive Sleep Apnea is Associated with Increased Arterial Stiffness

Longitudinal trials have shown that increased arterial stiffness (reduced arterial distensibility) precedes the development of essential hypertension and predicts future cardiovascular events among normotensive community-dwelling individuals. Asymptomatic arterial stiffening is an independent risk factor for essential hypertension and CVD. Insulin resistance has consistently been associated with increased arterial stiffness in a number of population groups, including the general population, patients with CKD, and patients with diabetes, independently of other vascular risk factors [85, 86, 87, 88, 89, 90]. Cross-sectional investigations associate OSA with increased arterial stiffness on different arterial areas, evaluated by a variety of procedures, including pulse wave velocity (carotid-femoral, brachial-ankle, or central), the augmentation index, the cardio-ankle vascular index, aortic distensibility calculated from the aortic diameters measured by echocardiography, ultrasound speckle-tracking-based analysis of carotid artery, and cardiovascular magnetic resonance of the aorta and carotid arteries. The association between OSA and arterial stiffening is independent of classical cardiovascular risk factors, such as systemic hypertension and general obesity (BMI). Increased arterial stiffness has been detected in normotensive patients with minimally symptomatic OSA, suggesting that subclinical vascular damage affects OSA patients from the onset of the disorder and precedes the development of arterial hypertension. In addition, OSA severity correlates with the degree of arterial stiffening [51, 53, 91, 92, 93, 94, 95, 96, 97, 98, 99, 100, 101, 102, 103, 104, 105, 106, 107, 108]. Systematic reviews and meta-analyses confirm that OSA is a risk factor for reduced arterial distensibility, independent of conventional cardiovascular factors [109, 110, 111]. Interventional studies show that therapy with positive air pressure (either continuous or autoadaptive) improves arterial stiffening in OSA patients compared to baseline values, ineffective CPAP or conservative therapy, supporting a role for tissue hypoxia in arterial stiffening. The improvement in arterial stiffness occurs early after the initiation of CPAP and reverts quickly with CPAP withdrawal [99, 112, 113, 114, 115, 116, 117, 118]. Systematic reviews and meta-analyses confirm that CPAP therapy reduces arterial stiffening in patients with OSA [109, 110, 119]. Likewise, OSA therapy with the implantation of a mandibular advancement device improves insulin resistance and arterial stiffening compared with baseline values [41].

#### 4.3.2 Obstructive Sleep Apnea is Associated with Essential Hypertension

The prevalence of essential hypertension is higher in patients with OSA (both children and adults) compared to control subjects, regardless of BMI and other risk factors. Further, OSA is associated with poorer blood pressure control among hypertensive patients. In addition, systolic and diastolic blood pressure values increase with OSA severity [4, 14, 26, 42, 43, 45, 46, 47, 49, 53, 54, 57, 60, 80, 120, 121, 122]. Longitudinal surveys on the general population [123] and the elderly [124] reveal that OSA precedes the development of systemic hypertension. Patients with OSA at baseline are at increased risk for developing essential hypertension at follow-up compared to control subjects without baseline OSA. In a systematic review and meta-analysis, pooled data from 6 studies show that OSA patients experience an increased risk for resistant hypertension compared to control subjects, after adjustment for traditional risk factors [125].

Notwithstanding the association between OSA and arterial hypertension, systematic reviews and meta-analyses of placebo-controlled randomized trials show that CPAP therapy reduces only slightly blood pressure values in OSA patients, the effect being more intense in patients with resistant hypertension [126, 127, 128, 129, 130, 131, 132, 133, 134, 135, 136, 137]. Similarly to CPAP, the implantation of mandibular advancement devices mildly reduces systolic and diastolic blood pressure [138].

#### 4.3.3 Obstructive Sleep Apnea is Associated with Left Ventricular Hypertrophy and Left Ventricular Dysfunction 

In 1990, a case-control investigation revealed that left ventricular hypertrophy (LVH) was more frequent in normotensive patients with OSA compared to control subjects without OSA. Both the left ventricular mass and the left ventricular mass index (left ventricular mass/body surface area) were larger among normotensive OSA patients, after adjustment for weight, suggesting that OSA is a risk factor for LVH even in normotensive patients [139]. Subsequent cross-sectional studies [50, 93, 140, 141, 142, 143, 144, 145, 146] and a meta-analysis [147] reveal that OSA patients (children and adults) are at increased risk for LVH, after controlling the confounding effects of hypertension, general obesity, and other factors. The prevalence of LVH in the pooled OSA population was 45% [147] while LVH has been reported in 88% of patients with severe OSA. Decreased aortic distensibility present in OSA patients increases left ventricular afterload and may contribute to an increase in left ventricular mass [141]. CPAP therapy in patients with OSA has been reported to reduce LVH compared to usual care [141, 148].

In addition to LVH, both children and adults with OSA may exhibit other echocardiographic abnormalities, including increased left ventricular diameters (diastolic and systolic), decreased left ventricular ejection fraction [149], systolic and diastolic left ventricular dysfunction (assessed by the left ventricle myocardial performance index) [150], left atrial enlargement [97, 141], right atrial enlargement, and right ventricular hypertrophy [140, 141].

#### 4.3.4 Obstructive Sleep Apnea is Associated with Increased Arterial Intima-Media Thickness

Cross-sectional and case-control trials show that OSA patients exhibit increased carotid IMT compared to control individuals. The severity of OSA correlates positively with carotid IMT. Increased carotid IMT is present in OSA patients without hypertension, diabetes or CVD, indicating that subclinical vascular injury occurs early in the evolution of the disorder, independent of traditional risk factors [50, 52, 64, 92, 98, 100, 151, 152, 153, 154, 155, 156]. A population-based, prospective cohort study that followed 790 randomly selected Wisconsin residents from a polysomnogram to a carotid ultrasound (average of 13.5 years) determined that OSA precedes thickening of the carotid artery. Baseline OSA predicted future increased carotid IMT independent of conventional cardiovascular risk factors [157]. In a systematic review and meta-analysis that included 18 studies, OSA was a risk factor for increased carotid IMT, after adjustment for major confounders. OSA patients had a higher carotid IMT compared to healthy controls [158]. CPAP therapy [159] or nasal surgery and uvulo-palato-pharyngoplasty (in OSA patients with narrowing at the nasal cavity and retropalatal airways) [160] have been reported to reduce carotid IMT compared to conservative treatment. However, no change of carotid IMT after CPAP treatment was observed in a meta-analysis that included 7 studies, although carotid IMT was decreased in patients with severe OSA who received CPAP ≥6 months [161].

#### 4.3.5 Obstructive Sleep Apnea is Associated with Reduced Endothelial Vasodilation

Cross-sectional and case-control investigations show that flow-mediated vasodilation and the reactive hyperemia index are reduced in OSA patients compared to control subjects, suggesting that OSA patients experience asymptomatic vascular damage [92, 95, 96, 115, 156]. In a systematic review and meta-analysis that included 18 articles, flow-mediated vasodilation in patients with OSA was lower compared to control subjects [111].

#### 4.3.6 Obstructive Sleep Apnea is Associated with Increased Clinical Cardiovascular Events

Cross-sectional [17, 42, 103, 162, 163] and longitudinal [164, 165, 166, 167, 168] studies reveal that the rate of clinical cardiovascular events (coronary artery disease, heart failure, peripheral artery disease, and stroke) is increased in OSA patients compared to control subjects. Longitudinal surveys have established that OSA precedes CVD. The diagnosis of OSA at baseline is a predictor of CVD independent of conventional cardiovascular risk factors [164, 165, 166, 167, 168]. Systematic reviews and meta-analyses consistently confirm that OSA is associated with increased cardiovascular risk [169, 170, 171, 172, 173, 174, 175, 176]. A beneficial effect of CPAP on cardiovascular outcomes compared to usual care has not been consistently found among OSA patients, particularly in randomized controlled clinical trials. In a recent systematic review and meta-analysis that included 11 studies (5 randomized controlled trials and 6 observational studies), CPAP therapy was associated with a modest risk reduction in cardiovascular events among patients with OSA and concomitant coronary artery disease. In addition, CPAP reduced the risk of all-cause and cardiovascular death by 23%. Subgroup analysis revealed that a CPAP adherence time ≥4 hours/night had a greater benefit on preventing cardiovascular events [137].

### 4.4 Obstructive Sleep Apnea is Associated with Impaired Glucose Tolerance and Increased Risk for Type 2 Diabetes

In OSA patients, insulin resistance causes impaired glucose tolerance and predisposes them to T2D. The prevalence of glucose intolerance is higher in OSA patients compared to control subjects, independent of BMI. Non-diabetic patients with OSA have increased levels of fasting glucose, postprandial glucose, and glycosylated hemoglobin, compared to control subjects, after adjustment for confounding factors [1, 21, 45, 46, 52, 60, 66, 80, 177, 178]. Likewise, cross-sectional [1, 43, 179, 180] and longitudinal [181, 182, 183] studies reveal an independent association between OSA and T2D. The prevalence of T2D is greater in OSA patients compared to control subjects, regardless of general obesity and other risk factors. Longitudinal investigations establish that OSA precedes T2D. Patients with OSA at baseline have a 30% higher risk of developing new-onset T2D at follow-up compared to control individuals, independent of age, race, gender, baseline fasting glucose, and BMI. A meta-analysis of 6 prospective cohort studies and 5953 participants confirms that OSA is an independent risk factor for the development of new-onset T2D, compared with the absence of OSA, over follow-up periods from 2.7 years to 16 years [184]. Longitudinal observational studies show that therapy with positive airway pressure [181, 185] or upper airway surgery [185] reduces the risk of new-onset T2D in OSA patients compared to conservative therapy, independent of confounding factors. Supporting the presence of more severe insulin resistance, T2D patients with OSA endure worse glycemic control compared to T2D patients without OSA, regardless of BMI. Even mild OSA has a negative influence on glycemic control among T2D patients [186, 187, 188]. Investigations on the effect of CPAP on glycemic control (glycosylated hemoglobin) in OSA patients with T2D have yielded inconsistent results. An association between hours of CPAP usage and glycosylated hemoglobin reduction has been observed, such that better adherence to CPAP is associated with greater improvements in glycemic control [189, 190].

### 4.5 Obstructive Sleep Apnea is Associated with Kidney Disease (Albuminuria, Glomerulomegaly, and Chronic Kidney Disease)

OSA-related insulin resistance has been associated with albuminuria, glomerulomegaly, CKD, and accelerated loss of kidney function toward end-stage kidney disease.

#### 4.5.1 Obstructive Sleep Apnea is Associated with Albuminuria

In 1984, a patient with severe OSA was noted to develop nephrotic syndrome [191]. Subsequent cross-sectional surveys showed that the prevalence of proteinuria (sometimes in nephrotic range) is higher in both children and adults with OSA compared to control subjects, after adjustment for confounding factors. Even OSA patients without diabetes or hypertension experience a higher risk of developing albuminuria (urinary albumin excretion rate or urinary albumin/creatinine ratio) compared to control subjects [192, 193, 194, 195, 196, 197, 198, 199, 200, 201]. In addition, an association between OSA severity and the degree of albuminuria has been observed [202, 203]. Systematic reviews and meta-analyses confirm that OSA is independently associated with higher urinary albumin excretion rate compared to control subjects [204, 205]. Therapy with positive airway pressure reduces albuminuria in OSA patients, either non-diabetic [192, 206, 207] or patients with diabetic kidney disease [208, 209]. CPAP effect on albuminuria is more profound in patients with more severe OSA [197].

#### 4.5.2 Obstructive Sleep Apnea is Associated with Glomerulomegaly and Focal Segmental Glomerulosclerosis 

OSA patients with proteinuria exhibit a kidney histopathological picture consistent with insulin resistance, namely glomerulomegaly, focal segmental glomerulosclerosis, and prominent involvement of kidney arterial vessels (hyaline sclerosis of arterioles and fibroelastic thickening of artery walls). The histopathological findings in OSA patients replicate those observed in other conditions associated with tissue hypoxia (and adaptive insulin resistance), such as high-altitude maladaptation, sickle cell disease, and cyanotic congenital heart disease, suggesting a causative role for hypoxia-related insulin resistance in the kidney lesion [206, 210, 211]. On light microscopy, glomeruli are markedly enlarged and focal segmental glomerulosclerosis is observed. Mesangial matrix and mesangial cellularity are usually increased. Arterial involvement is conspicuous with marked arteriolar hyalinosis and arterial intima-media thickening. Correspondingly with arterial damage, focal areas of tubular atrophy and interstitial fibrosis are identified. Immunofluorescence staining is usually negative. Electron microscopy reveals effacement of podocyte foot processes. No subendothelial, mesangial or subepithelial immune complex deposits are noted [206, 210, 211].

#### 4.5.3 Obstructive Sleep Apnea is Associated with Chronic Kidney Disease

OSA-related insulin resistance is an independent risk factor for CKD and accelerated progression to end-stage kidney disease [212]. The prevalence of CKD, defined as estimated glomerular filtration rate (GFR) <60 mL/min/1.73 $m^{2}$, is higher among OSA patients compared to control subjects, after adjustment for BMI and other confounders. GFR estimation was assessed with the MDRD (modification of diet in renal disease) equation, the CKD-EPI (CKD Epidemiology Collaboration) equation, or the Cockcroft-Gault formula, the prevalence of CKD remains higher in OSA patients without essential hypertension or T2D. A positive correlation between OSA severity and worse kidney function has been found [200, 201, 202, 213, 214, 215, 216, 217, 218, 219]. Longitudinal studies show that OSA is associated with an increased risk for CKD and end-stage kidney disease compared with the general population, independent of conventional risk factors [212, 220, 221, 222]. In addition, cross-sectional [219] and longitudinal [223, 224] investigations show that OSA patients experience accelerated loss of kidney function, compared to control subjects, after adjustment of risk factors such as age, BMI, diabetes and heart failure. The loss of kidney function is faster in participants with OSA over the follow-up period. Systematic reviews and meta-analyses confirm that OSA patients endure a higher risk of CKD (lower estimated GFR), compared to control subjects. Accordingly, the serum level of cystatin C is increased in OSA patients compared to control individuals, independent of hypertension and diabetes [204, 205]. Likewise, OSA is associated with diabetic kidney disease in T2D patients [225]. Investigations concerning the effect of CPAP therapy on kidney function have rendered conflicting results. Some studies show a slower rate of progression among OSA patients treated with CPAP [226, 227, 228] whereas a systematic review and meta-analysis found no clinically relevant effect of CPAP on estimated GFR [229].

## 5. Oxyhemoglobin Desaturation (Leading to Tissue Hypoxia) Induces Insulin Resistance in Patients with Obstructive Sleep Apnea 

In humans, insulin resistance is an adaptive response to normobaric hypoxia [230, 231, 232, 233, 234] and hypobaric hypoxia (exposure to high altitude) [235, 236, 237]. Likewise, oxyhemoglobin desaturation (leading to tissue hypoxia) has been consistently linked to insulin resistance and its clinical consequences in OSA patients. The severity of oxygen desaturation in these patients may be expressed in a variety of ways, such as the average nocturnal oxygen saturation, the lowest oxygen saturation, the percentage of time spent below 90% oxygen saturation during sleep, and the oxyhemoglobin desaturation index, defined as the average number of desaturation episodes occurring per hour. A desaturation episode is a reduction in oxyhemoglobin saturation ≥3% that lasts ≥10 seconds. Oxyhemoglobin saturation is the percentage of hemoglobin bound to oxygen.

### 5.1 Oxyhemoglobin Desaturation is Independently Associated with Insulin Resistance in Patients with Obstructive Sleep Apnea

Cross-sectional studies show that oxyhemoglobin desaturation is strongly and independently associated with insulin resistance in children and adults with OSA [1, 2, 3, 4, 6, 8, 9, 10, 11, 23, 56, 62, 177, 238, 239, 240, 241]. Likewise, a prospective community-based study with a mean follow-up period of 11 years reveals that more severe oxygen desaturation at baseline is independently associated with more profound insulin resistance at follow-up (evaluated by the HOMA-IR index and the insulin sensitivity index) [32].

### 5.2 Oxyhemoglobin Desaturation is Independently Associated with the Metabolic Syndrome and its Components in Patients with Obstructive Sleep Apnea

OSA patients with lower oxyhemoglobin saturation experience clinical manifestations of insulin resistance, regardless of BMI and other risk factors. A variety of oxygen desaturation estimates (oxyhemoglobin desaturation index, minimum arterial oxygen saturation, and mean nocturnal oxygen saturation) are independently and robustly associated with metabolic syndrome [49], visceral adiposity [55, 59, 62], non-alcoholic fatty liver disease [242], epicardial fat thickness [77], and dyslipidemia (hypertriglyceridemia and decreased HDL-c) [4, 25, 48, 55, 80, 82] among both obese and non-obese OSA patients.

### 5.3 Oxyhemoglobin Desaturation is Independently Associated with Cardiovascular Disease in Patients with Obstructive Sleep Apnea

In OSA patients, the hypoxia burden is correlated with subclinical vascular damage. The lowest oxygen saturation, the average oxygen saturation, the oxyhemoglobin desaturation index, and the percentage of time spent below 90% oxygen saturation during sleep, are robustly and independently associated with increased arterial stiffness [96, 243, 244], elevated blood pressure [4, 48, 49, 120, 245], increased carotid IMT [98, 152, 153, 155, 246], and left ventricular dysfunction [146, 245, 247, 248, 249]. Unlike the apnea hypopnea index, oxyhemoglobin desaturation strongly predicts asymptomatic vascular injury and myocardial performance in OSA patients, after adjustment for conventional cardiovascular risk factors. Accordingly, the improvement in oxygen saturation after uvulo-palato-pharyngoplasty showed a correlation with a reduction of arterial IMT [160].

### 5.4 Oxyhemoglobin Desaturation is Associated with Impaired Glucose Tolerance, Worse Glycemic Control, and Increased Risk of Type 2 Diabetes in Patients with Obstructive Sleep Apnea

OSA patients with more severe nocturnal hypoxemia (assessed by the oxyhemoglobin desaturation index) show an increased risk of T2D [187] and more elevated fasting glucose levels [48, 80], after adjustment for obesity and other confounding factors. In addition, OSA patients with T2D and more pronounced hypoxemia endure worse metabolic control. The percentage of time spent under 90% oxygen saturation and the minimum oxygen saturation are associated with higher glycosylated hemoglobin (HbA1c), independent of general adiposity and other confounders. There is a positive relationship between nocturnal hypoxemia and deficient glycemic control in OSA patients with T2D. Every 10% reduction in minimum oxygen saturation is associated with a 0.3% increase in HbA1c whereas a 10% increase in the percentage of time spent under 90% oxygen saturation is associated with a 0.2% increase in HbA1c [186, 187, 188, 250]. Longitudinal studies reveal that decreased oxygen saturation at baseline predicts incident T2D at follow-up in OSA patients, after adjustment for BMI and other risk factors, suggesting that more severe hypoxemia drives insulin resistance and predisposes to T2D. Unlike the apnea-hypopnea index, the time spent with oxygen saturation of less than 90% is associated with incident diabetes in adjusted models [1, 32, 56, 181, 182, 183].

### 5.5 Oxyhemoglobin Desaturation is Independently Associated with Kidney Disease in Patients with Obstructive Sleep Apnea

In OSA patients, a more severe hypoxia burden has been consistently associated with increased albuminuria and worse kidney function. Children and adults with more pronounced nocturnal hypoxemia (assessed by the lowest oxygen saturation, the length of sleep time spent at oxygen saturation below 90%, and the desaturation index) experience more severe albuminuria, independent of BMI and other confounders [195, 196, 197, 198, 200, 214, 251, 252]. Accordingly, proteinuria falls after improvement of oxygen saturation with CPAP [252, 253]. The prevalence of CKD (estimated GFR <60 mL/min/1.73 $m^{2}$) is higher in OSA patients with more severe hypoxia burden, after adjusting for confounders. Profounder nocturnal hypoxemia (evaluated by the time spent under 90% oxygen saturation, the mean oxygen saturation, and the lowest nocturnal oxygen saturation) is inversely correlated with estimated GFR, such that OSA patients with more severe hypoxia burden experience worse kidney function (lower estimated GFR). Furthermore, the minimum oxygen saturation is an independent predictor of CKD, indicating that severe hypoxemia, even for a short period of time, is a risk factor for kidney dysfunction [200, 216, 217, 227]. Retrospective cohort trials reveal that oxyhemoglobin desaturation is independently associated with an increased risk for accelerated loss of kidney function and progression to end-stage kidney disease. Patients with more severe hypoxemia experience a faster loss of kidney function [223, 224]. Likewise, more profound nocturnal hypoxemia (time spent under 90% oxygen saturation) is associated with diabetic kidney disease and worse kidney function, after adjusting for confounding variables in patients with OSA and T2D [225, 254]. Accordingly, CPAP therapy improves oxyhemoglobin desaturation and decelerates the rate of CKD progression in patients with CKD and OSA [227]. In OSA patients with advanced CKD, more severe hypoxemia (higher sleep time with oxygen saturation below 90% and lower mean oxygen saturation) is associated with higher mortality over the follow-up period (9 years) [255].

## 6. Pathophysiological Processes Underlying Insulin Resistance in Patients with Obstructive Sleep Apnea

Pathogenic mechanisms leading to adaptive insulin resistance due to tissue hypoxia in OSA patients involve the upregulation of HIFs and downregulation of PPAR-γ. Silencing of PPAR-γ in adipocytes reduces glucose uptake, impairs lipid droplet biogenesis, and induces whole-body insulin resistance [234].

### 6.1 Hypoxia Induces HIF-1α Upregulation and Subsequent PPAR-γ Downregulation in Patients with Obstructive Sleep Apnea

**Fig. 2.**
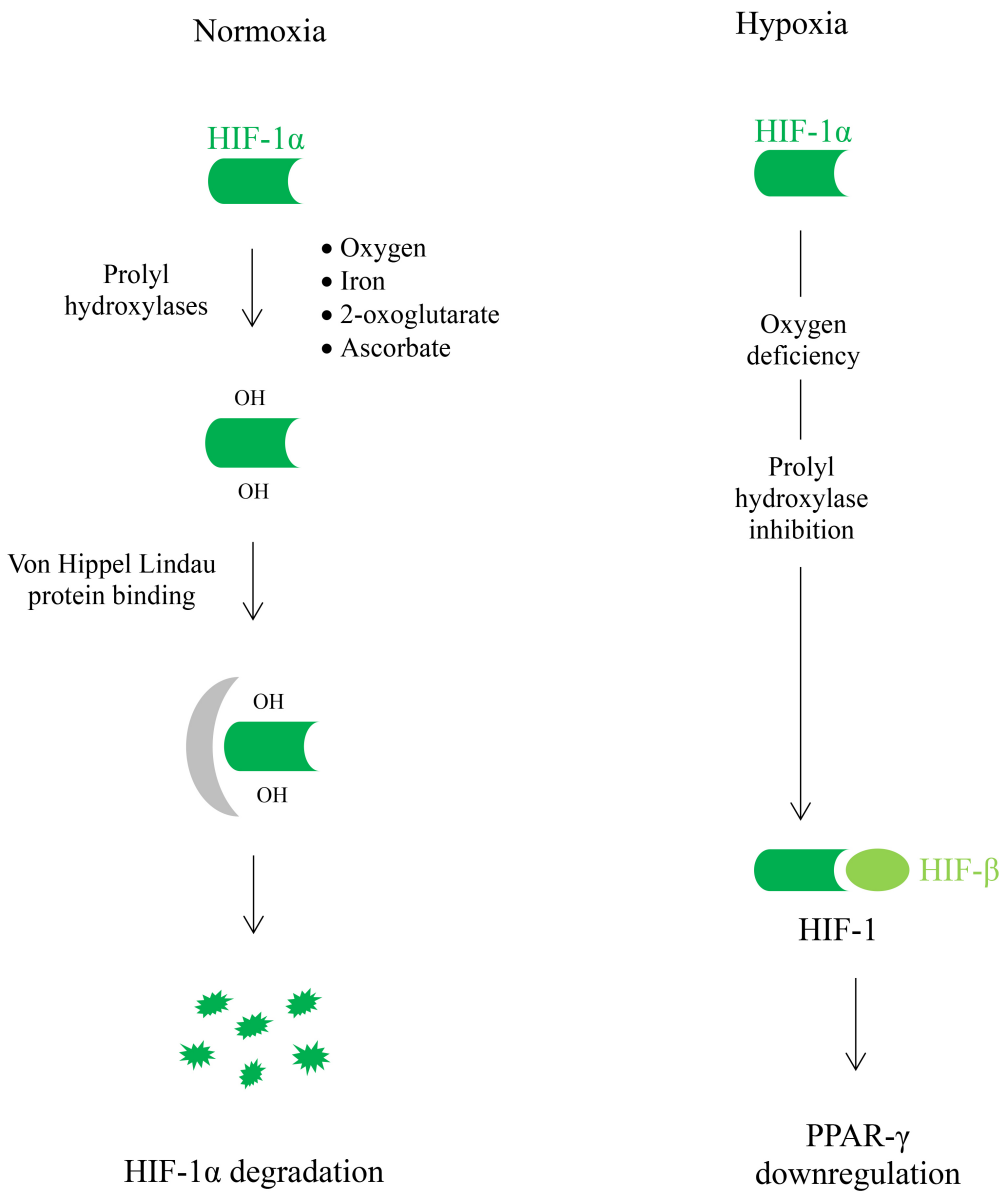
**In humans, formation of human hypoxia-inducible factors (HIFs) can occur only under hypoxia.** Under normal conditions (normoxia), HIF-α is continuously degraded and cannot dimerize with HIF-β. Breakdown of HIF-α requires the action of prolyl hydroxylase isoenzymes and the subsequent attachment to the von Hippel Lindau protein, which labels HIF-α for degradation. HIF-α prolyl hydroxylase isoenzymes require oxygen to catalyze HIF-α hydroxylation. Therefore, hypoxia inhibits this reaction, impedes HIF-α degradation and allows the formation of functional HIF-1. Once activated, HIF-1α downregulates the expression of *PPARG* gene, which codes for peroxisome proliferatoractivated receptor gamma (PPAR-γ) and suppresses PPAR-γ activity.

HIFs are transcription factors that regulate the transcription of target genes by binding to specific DNA sequences named hypoxia response elements. HIFs are heterodimeric proteins that possess two subunits, α and β. Three isoforms of the α subunit have been identified (HIF-1α, HIF-2α, and HIF-3α) whereas only one β subunit has been reported to exist in humans. Each isoform of the α subunit may dimerize with the β subunit to generate the corresponding HIF (HIF-1, HIF-2, or HIF-3). Formation of human HIFs can only be completed when the oxygen supply to tissues is limited, because, under normoxia, HIF-α is continuously degraded and unavailable for dimerization with HIF-β. Degradation of HIF-α requires initial hydroxylation (catalyzed by prolyl hydroxylase isoenzymes) and subsequent binding to the von Hippel Lindau protein. The hydroxylation of specific proline residues in HIF-α permits the binding to the von Hippel Lindau protein. In turn, attachment to the von Hippel Lindau protein labels hydroxylated HIF-α to be destroyed [256, 257]. Therefore, inhibition of HIF-α prolyl hydroxylases and/or a defective von Hippel Lindau protein block the degradation of HIF-α and enable the formation of functional HIFs in humans [258]. HIF-α prolyl hydroxylase isoenzymes require oxygen, 2-oxoglutarate, iron, and ascorbate for activity. Consequently, deficiency of either one of them inhibits the hydroxylation of HIF-α, blocks its degradation, and allows the dimerization of intact HIF-α with the β subunit to produce operative HIFs [256, 257] (Fig. 2). Oxygen deprivation (hypoxia) inhibits the activity of prolyl-hydroxylases, promoting the formation of HIFs. *In vitro* studies using a variety of human cell lines show that HIF-1α expression increases during hypoxia exposure [259, 260, 261, 262, 263, 264]. Similarly to oxygen shortage, the deficiency of 2-oxoglutarate [265], iron [266], and ascorbate [267] suppresses HIF-α prolyl hydroxylases and generates HIFs. Once activated, HIFs modulate the transcription of target genes to orchestrate the response to hypoxia or other conditions. The *PPARG* gene, which codes for PPAR-γ, has been identified as a target gene for HIF-1 in human cells, including adipocytes. In response to hypoxia, HIF-1 reduces *PPARG* expression (mRNA and protein) and inhibits PPAR-γ activity [259, 260, 263, 268, 269, 270, 271].

### 6.2 Patients with Obstructive Sleep Apnea Exhibit HIF1A Upregulation and PPARG Downregulation in the Adipose Tissue

In OSA patients, increased expression of the *HIF1A* gene (which encodes HIF-1α) and downregulation of *PPARG* have been observed in the subcutaneous adipose tissue, compared to control subjects, independent of BMI [5, 272]. Additionally, OSA patients demonstrate higher serum levels of HIF-1α compared with control subjects. Further, serum HIF-1α level correlates with the number of desaturations during sleep [273].

### 6.3 PPAR-γ Silencing Causes Insulin Resistance Due to Defective Glucose Uptake into Adipocytes Leading to Suppression of Adipogenesis

**Fig. 3.**
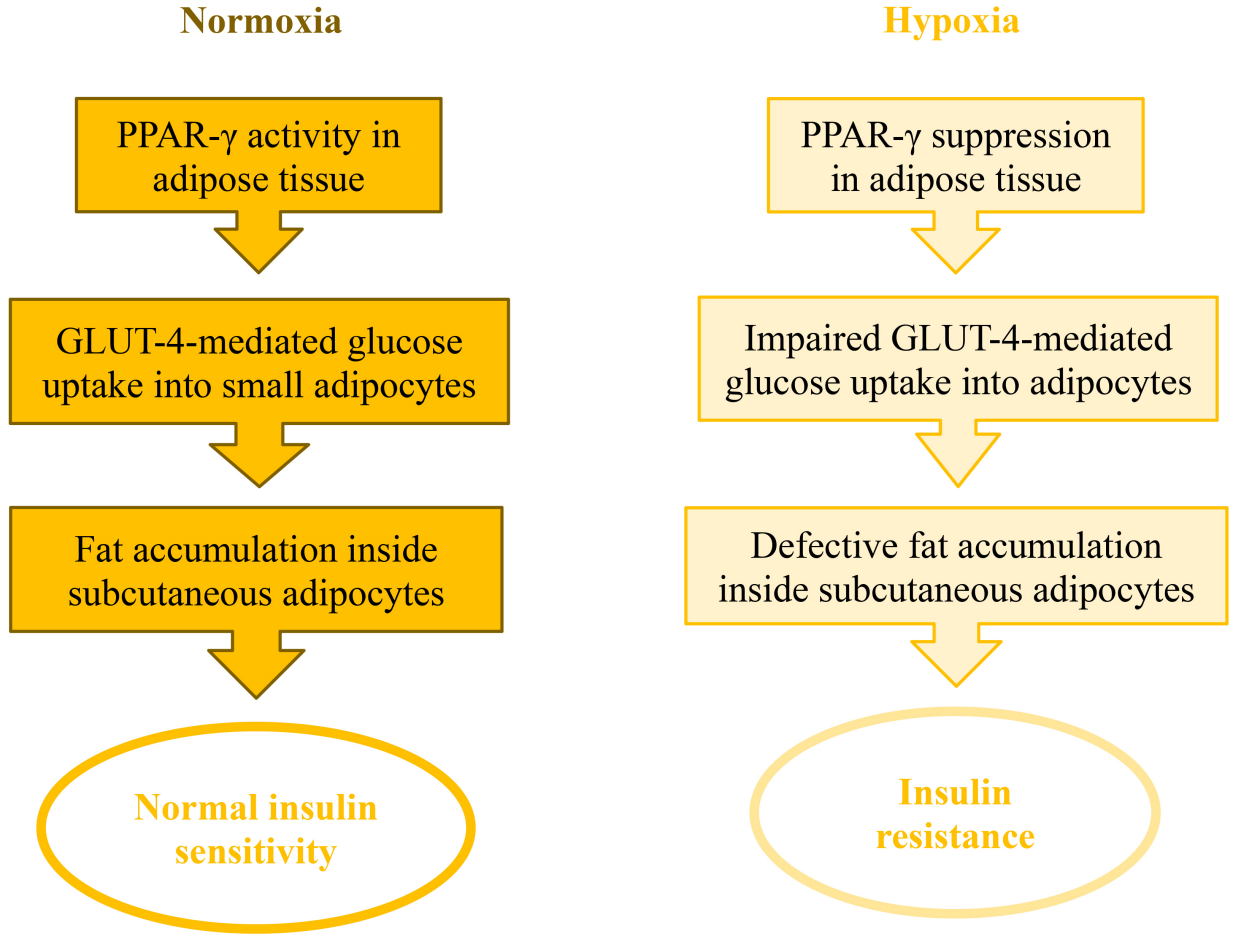
**In humans, normal PPAR-γ activity in adipocytes involves glucose uptake mediated by the glucose transporter-4 (GLUT-4) and fat accumulation in the subcutaneous adipose tissue while enhancing whole-body insulin sensitivity.** PPAR-γ silencing during hypoxic conditions induces the opposite effects, namely reduction of GLUT-4-mediated glucose transport into adipocytes, suppression of fat deposition, and whole-body insulin resistance. PPAR-γ, peroxisome proliferatoractivated receptor gamma.

In humans, glucose uptake into adipocytes by the glucose transporter-4 (GLUT-4) facilitates lipid droplet biogenesis, fat accumulation in the subcutaneous adipose tissue, and whole-body insulin sensitivity. These processes are accomplished by normal PPAR-γ activity. Thiazolidinediones (exogenous PPAR-γ agonists) replicate PPAR-γ effects whereas PPAR-γ silencing (such as occurs due to hypoxia-induced HIF-1α activation) mediates the opposite effects, namely reduction of GLUT-4-mediated glucose transport into adipocytes, suppression of fat deposition and lipid droplet formation in the subcutaneous adipose tissue, and whole-body insulin resistance (Fig. 3).

#### 6.3.1 Glucose Uptake into Adipocytes Promotes Adipocyte Differentiation and Defines Whole-Body Insulin Sensitivity in Normal Humans

6.3.1.1 Glucose Uptake into Adipocytes Mediated by the Glucose Transporter-4 Promotes Lipid Droplet Formation and Fat Deposition in the Adipose Tissue

In normal humans, GLUT-4 proteins carry glucose into adipocytes. The quantity of GLUT-4 declines markedly when adipocytes enlarge due to fat deposition, regardless of BMI and degree of insulin sensitivity, such that large adipocytes contain strikingly fewer GLUT-4 molecules compared to small adipocytes in lean individuals, obese subjects, and T2D patients. Correspondingly, GLUT-4 conveys glucose into small adipocytes while no relevant transport is detected on large cells from human omental and subcutaneous adipose tissue, suggesting that glucose uptake into small adipocytes has a crucial effect on triacylglycerol deposition and subsequent adipocyte enlargement, that is no longer required in large adipocytes [274, 275, 276, 277].

6.3.1.2 The Expression of Glucose Transporter-4 in Adipocytes Correlates with Whole-Body Insulin Sensitivity

The amount of GLUT-4 in small adipocytes correlates with whole-body insulin sensitivity, measured by euglycemic hyperinsulinemic clamps, such that subjects with enhanced insulin sensitivity possess more GLUT-4 whereas patients with insulin resistance possess markedly diminished GLUT-4 (mRNA and protein) levels in small adipocytes. Correspondingly, GLUT-4-mediated glucose uptake into subcutaneous adipocytes is markedly reduced in patients with insulin resistance (including T2D), suggesting that defective glucose uptake into subcutaneous adipocytes contributes to cause whole-body insulin resistance [276, 277, 278, 279, 280, 281, 282, 283].

Similarly to other subjects with insulin resistance, patients with gestational diabetes [284, 285] or polycystic ovary syndrome [286, 287, 288] display reduced GLUT-4 expression and impaired glucose uptake into adipocytes compared to control subjects, independent of obesity. The amount of GLUT-4 in adipocytes is highly correlated with the degree of insulin sensitivity among these patients, such that abundance of GLUT-4 is associated with enhanced insulin sensitivity while scarcity of GLUT-4 is observed in patients with insulin resistance (assessed by insulin-mediated glucose disposal or HOMA-IR index). In a systematic review of the literature, decreased GLUT-4 content in adipocytes (and subsequent impaired glucose transport) is a consistent abnormality in patients with polycystic ovary syndrome, regardless of general adiposity [288].

6.3.1.3 A Reduced Amount of GLUT-4 in Adipocytes and Reduced Fat Accumulation in the Subcutaneous Adipose Tissue Occur at the Onset of Insulin Resistance

Subjects with normal weight, glucose tolerance, and fasting triglyceride level, but with a genetic predisposition for T2D (first-degree relatives of T2D patients) demonstrate a profound reduction of GLUT-4 (mRNA and protein) in adipocytes [282, 289]. The magnitude of GLUT-4 reduction in subcutaneous adipocytes among these subjects is quantitatively similar to that observed in patients with T2D or impaired glucose tolerance [280]. In addition, healthy first-degree relatives of T2D patients exhibit a markedly diminished capability to store fat in the subcutaneous adipose tissue leading to an inappropriate expansion of the adipose cells. In these subjects, insulin resistance is identified by euglycemic hyperinsulinemic clamps [282, 289]. Therefore, reduced GLUT-4 in adipocytes and an impaired ability to store fat in the subcutaneous adipose tissue precedes any other clinical manifestation of insulin resistance, suggesting that these metabolic abnormalities occur at the onset of this metabolic adaptation [289].

6.3.1.4 *In vitro* Studies Indicate that Glucose Uptake into Adipocytes is Required for Fat Accumulation and Lipid Droplet Biogenesis

The link between glucose uptake into adipocytes and the ability to accumulate fat is further supported by *in vitro* studies using primary human adipocytes. Silencing of an endoplasmic reticulum protein involved in lipid droplet biogenesis (fat storage-inducing transmembrane protein-2) in primary human adipocytes reduces both glucose uptake and triacylglycerol accumulation, compared with control cells suggests that glucose transport into adipocytes is required for fat accumulation [290]. Consistently, fat storage-inducing transmembrane protein-2 is less abundant in the subcutaneous and omental adipocytes from T2D patients, compared to control subjects [290].

#### 6.3.2 Human PPAR-γ Facilitates Glucose Uptake into Adipocytes, Subcutaneous Fat Deposition, and Insulin Sensitivity while PPAR-γ Inhibition has the Converse Effect

In normal humans, PPAR-γ normally promotes glucose uptake into adipocytes and triglyceride deposition on the subcutaneous adipose tissue while enhancing insulin sensitivity. PPAR-γ activity is essential for the process of subcutaneous fat deposition and adipocyte differentiation, but is not required for maintenance of the differentiated state. Depletion of PPAR-γ prevents fat deposition inside subcutaneous adipocytes but does not induce dedifferentiation of mature adipocytes once full differentiation has taken place. In OSA patients, PPAR-γ suppression is associated with decreased glucose uptake into adipocytes, reduced fat accumulation in the subcutaneous adipose tissue, and whole-body insulin resistance [291, 292, 293]. Thiazolidinediones (such as pioglitazone and rosiglitazone) are exogenous PPAR-γ ligands that activate PPAR-γ and reproduce its effects. In non-diabetic patients with insulin resistance (and therefore low GLUT-4 protein in adipose cells), pioglitazone therapy increases GLUT-4 expression in subcutaneous adipose tissue (mRNA and protein) and improves insulin resistance (assessed by euglycemic hyperinsulinemic clamps), strengthening the existence of a mechanistic link between reduced GLUT-4 protein in adipocytes and whole-body insulin resistance [282].

The study’s strengths include a meticulous and unbiased literature search attempting to retrieve all relevant articles on the connection between insulin resistance and OSA. However, some limitations need to be acknowledged. First, we searched only the PubMed database and restricted our search to articles published in English. As a result, some degree of selection bias may have occurred. Second, included studies were heterogenous in terms of study design, classifications for the severity of OSA, population groups, duration of follow-up, and the method of assessing complications of insulin resistance (for instance, blood pressure recordings, arterial stiffness, and kidney function). In addition, except for articles concerning OSA therapy, included investigations were mostly observational, limiting their ability to demonstrate causality.

## 7. Conclusions

Patients with obstructive sleep apnea experience apnea episodes that induce oxyhemoglobin desaturation and subsequent tissue hypoxia. In turn, tissue hypoxia induces adaptive insulin resistance. In patients with obstructive sleep apnea, oxyhemoglobin desaturation has been consistently associated with insulin resistance and its clinical consequences, independent of body mass index and other confounding factors. Patients with obstructive sleep apnea typically manifest metabolic syndrome, essential hypertension, hypertriglyceridemia, reduced HDL-c, visceral adiposity, non-alcoholic fatty liver disease, increased epicardial fat, subclinical vascular injury (such as reduced arterial distensibility, left ventricular hypertrophy, and increased arterial intima-media thickness), elevated cardiovascular risk, impaired glucose tolerance, predisposition to type 2 diabetes, and kidney disease (glomerulomegaly, albuminuria, and chronic kidney failure). In patients with obstructive sleep apnea, tissue hypoxia upregulates hypoxia-inducible factor-1, which induces peroxisome proliferator-activated receptor-gamma downregulation in the adipose tissue. Attenuation of peroxisome proliferator-activated receptor-gamma reduces glucose transport into adipocytes, impairs fat deposition in the subcutaneous adipose tissue and causes whole-body insulin resistance. Obesity is a major causative factor for obstructive sleep apnea, but insulin resistance develops in these patients irrespective of general adiposity (body mass index). Tissue hypoxia drives insulin resistance in patients with obstructive sleep apnea.

## Abbreviations

BMI, body mass index; CKD, chronic kidney disease; CPAP, continuous positive airway pressure; CVD, cardiovascular disease; GFR, glomerular filtration rate; GLUT, glucose transporter; HDL-c, cholesterol associated with high density lipoprotein; HIF, hypoxia-inducible factor; HOMA-IR, homeostasis model assessment-insulin resistance; IMT, intima-media thickness; LVH, left ventricular hypertrophy; NAFLD, non-alcoholic fatty liver disease; OSA, obstructive sleep apnea; PPAR, peroxisome proliferator-activated receptor.
